# The complete chloroplast genome of *Cuscuta australis* R. Br. (*Convolvulaceae*) and its phylogenetic implication

**DOI:** 10.1080/23802359.2020.1715865

**Published:** 2020-01-22

**Authors:** Fei-fei Wang, Dao-jun Huang, Bao-li Qin, Xia-wen Wang, Qian Jin, Zhi-hui Zhang, Xin-hai Wang

**Affiliations:** Suqian Institute of Agricultural Sciences, Jiangsu Academy of Agricultural Sciences, Suqian, China

**Keywords:** *Cuscuta australis*, complete chloroplast genome, phylogenetic analysis, *Convolvulaceae*

## Abstract

The genus *Cuscuta* (*Convolvulaceae*) is an annual parasitic twining herb. There are about 200 species in this genus, which are widely distributed in tropical and subtropical areas. *Cuscuta* is mainly parasitic on crops bringing significant losses to the production of agriculture. Furthermore, dried seeds of *C. chinensis* and *C. australis* are used as a Chinese traditional herbal medicine. Despite the importance of *Cuscuta* species, it is difficult to distinguish these plants by the naked eye. Moreover, plastid sequence information available for *Cuscuta* species is limited. In this study, the complete chloroplast (cp) genome sequence of *C. australis* was determined using next-generation sequencing. The entire cp genome was determined to be 85,263 bp in length. It contained large single-copy (LSC) and small single-copy (SSC) regions of 50,384 and 6727 bp, respectively, which were separated by a pair of 14,076 bp inverted repeat (IR) regions. The genome contained 98 genes, including 61 protein-coding genes, 29 tRNA genes, and 4 rRNA genes. The overall GC content of the genome is 37.8%. A phylogenetic tree reconstructed by 26 chloroplast genomes reveals that *C. australis* is most related with *Cuscuta pentagona* in *Convolvulaceae*, with bootstrap support values of 100%.

The genus *Cuscuta* comprises about 200 well-known parasitic plants, which are widely distributed in tropical and subtropical areas (Costea et al. 2015). *Cuscuta* is mainly parasitic on crops bringing significant losses to the production of agriculture and garden industry (Zhang et al. 2009). Furthermore, dried seeds of *C. chinensis* and *C. australis* are used as a Chinese traditional herbal medicine. Despite the importance of *Cuscuta* species, it is difficult to distinguish these plants by the naked eye. Moreover, chloroplast genome of *C. australis* has not been reported. So, it is necessary to develop genomic resources for *C. australis* to provide basic intragenic information for the further study on phylogeny for genus *Cuscuta.*

The total genomic DNA was extracted from the fresh leaves of *C. austr*alis (N32°06′, E118°37′) using the DNeasy Plant Mini Kit (Qiagen, Valencia, CA, USA). The voucher specimen was deposited at the Herbarium of Suqian Institute of Agricultural Sciences (NFTS: 190711). The whole genome sequencing was conducted on the Illumina Hiseq 4000 Sequencing System (Illumina, Hayward, CA). The filtered sequences were assembled using the program SPAdes assembler 3.10.0 (Bankevich et al. 2012). Annotation was performed using the DOGMA (Wyman et al. 2004). All the tRNA sequences were confirmed using the web-based online tool, tRNAScan-SE (Schattner et al. 2005) with default settings to corroborate tRNA.

The plastome of *C. australis* was determined to comprise double-stranded, circular DNA of 85,263 bp containing two inverted repeat (IR) regions of 14,076 bp each, separated by large single-copy (LSC) and small single-copy (SSC) regions of 50,384 and 6,727 bp, respectively (NCBI acc. no. MN866891). The genome contained 98 genes, including 61 protein-coding genes, 29 tRNA genes, and four rRNA genes. The two protein-coding genes, four tRNA genes, and four rRNA genes were duplicated in IR region. Five genes (rps12, rps12, petB, petD, rps16) contained two exons and one gene (clpP) contained three exons. The overall GC content of *C. australis* cp genome is 37.8% and the corresponding values in LSC, SSC, and IR regions are 35.9, 30.0, and 43.2%, respectively.

To investigate its taxonomic status, whole chloroplast genomes from 24 *Convolvulaceae* plants and two outgroup plants (*Nicotiana tabacum* and *Capsicum annuum*) were aligned by MAFFT version 7 (Katoh and Standley 2013). A maximum likelihood (ML) was reconstructed based on FastTree version 2.1.10 (Price et al. 2010) (Figure 1). The ML phylogenetic tree shows that *C. australis* is most related with *Cuscuta pentagona* in *Convolvulaceae*, with bootstrap support values of 100%.

**Figure 1.**
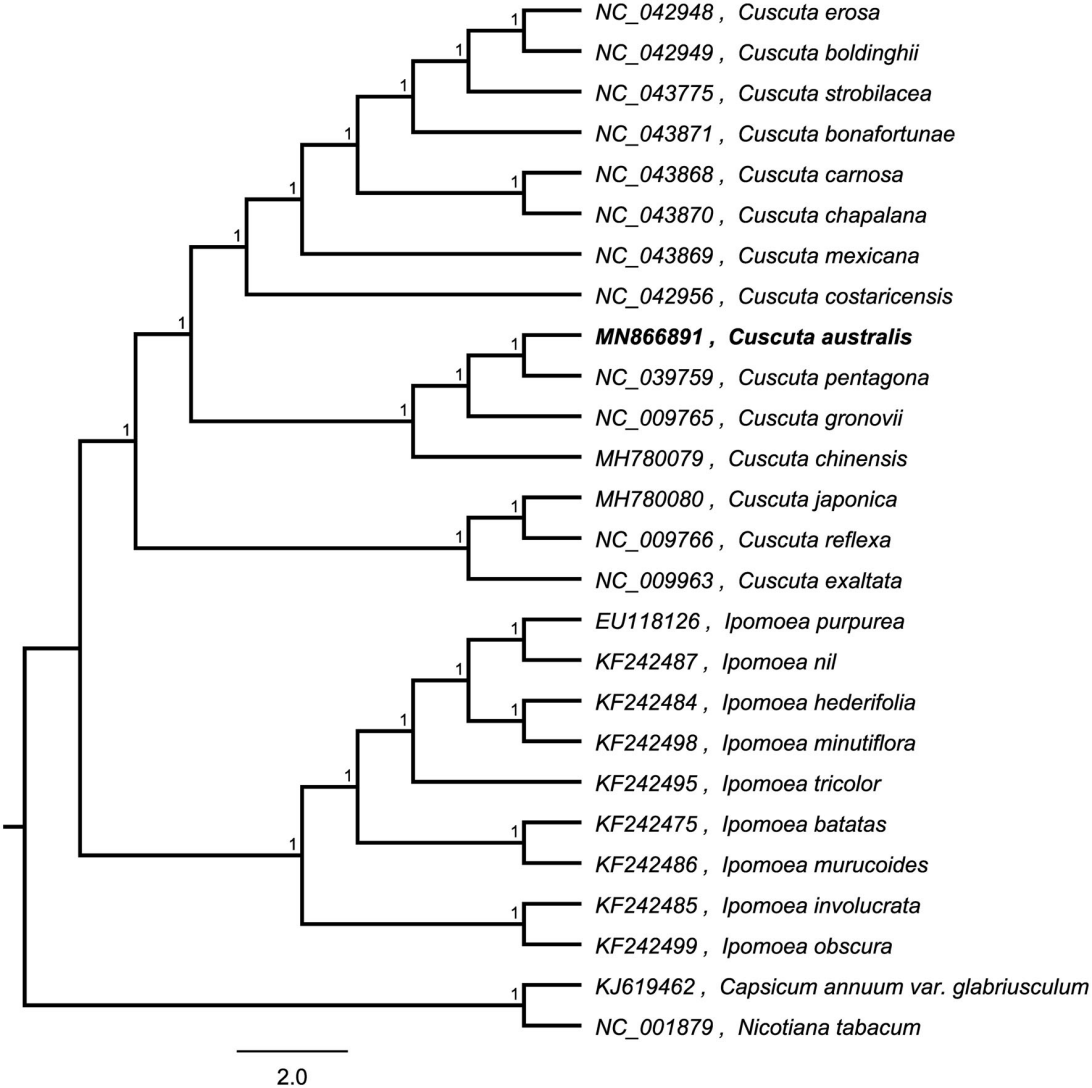
Maximum-likelihood phylogenetic tree based on whole chloroplast genomes from 24 *Cucumis* plants and two outgroup plant (*Nicotiana tabacum* and *Capsicum annuum*) and the support values are shown at the branches.
